# GFP nanobodies reveal recently-exocytosed pHluorin molecules

**DOI:** 10.1038/s41598-019-44262-8

**Published:** 2019-05-23

**Authors:** Katharina J. Seitz, Silvio O. Rizzoli

**Affiliations:** 1 Institute for Neuro- and Sensory Physiology, University Medical Center, Göttingen, Germany; 2 Center for Biostructural Imaging of Neurodegeneration, University Medical Center Göttingen, Göttingen, Germany

**Keywords:** Vesicle trafficking, Fluorescence imaging

## Abstract

Neurotransmitter release requires vesicle recycling, which consists of exocytosis, endocytosis and the reformation of new fusion-competent vesicles. One poorly understood aspect in this cycle is the fate of the vesicle proteins after exocytosis, when they are left on the plasma membrane. Such proteins are often visualized by coupling to pH-sensitive GFP moieties (pHluorins). However, pHluorin imaging is typically limited by diffraction to spots several-fold larger than the vesicles. Here we show that pHuorin-tagged vesicle proteins can be easily detected using single-domain antibodies (nanobodies) raised against GFP. By coupling the nanobodies to chemical fluorophores that were optimal for super-resolution imaging, we could analyze the size and intensity of the groups of pHluorin-tagged proteins under a variety of conditions, in a fashion that would have been impossible based solely on the pHluorin fluorescence. We conclude that nanobody-based pHluorin detection is a promising tool for investigating post-exocytosis events in neurons.

## Introduction

Synaptic vesicles fuse to the plasma membrane of the presynaptic bouton, and release thereby their neurotransmitter contents (exocytosis). This is followed by the retrieval of the vesicle proteins (endocytosis), and by the reformation of the new vesicle. These processes, termed altogether the “vesicle cycle”^1^ have been studied for several decades, and a large number of models have been proposed for different aspects of the cycle. For example, multiple models have been introduced for the synaptic vesicle endocytosis, from clathrin-mediated retrieval directly at the plasma membrane to ultrafast endocytosis, which is then followed by clathrin-dependent mechanisms inside the synapse, on endosome-like organelles^2,3^. As another example, the involvement of *bona fide* endosome sorting in the synaptic vesicle cycle remains unclear^4,5^.

Another issue still under discussion is the organization of the synaptic vesicle molecules during and after exocytosis, before endocytosis. Two main models have been proposed for this process^1,6,7^. In the first model, the vesicle proteins maintain to some extent their relative locations after fusion to the plasma membrane, as a patch of vesicle molecules. This patch would be presumably then identified by the endocytosis machinery, before being retrieved. In a second model, the vesicle proteins separate from each other after fusion, and the different proteins diffuse in the synapse plasma membrane, before being endocytosed, presumably in the form of assemblies of plasma membrane and vesicle molecules.

These scenarios have been difficult to test directly, but substantial information has been gathered by immunostaining the neuronal plasma membrane using antibodies directed against intraluminal epitopes of synaptic vesicle proteins, which are only exposed after exocytosis, such as the intravesicular domain of the calcium sensor synaptotagmin 1 (see for example^5,8,9^). These antibodies typically revealed groups of vesicle molecules on the plasma membrane, suggesting a limited segregation of vesicle proteins after exocytosis. However, this approach is not free of potential controversies. Synaptotagmin 1 antibodies have been in use for more than two decades^10,11^ and they do not appear to impair synaptic function^8,12^, but a significant modulation of vesicle recycling via the antibodies has been recently noted^13^. Thus, the usage of such antibodies is likely to provide a realistic image of the vesicle protein organization after exocytosis, but may have some modulatory side effects. At the same time, since antibodies are bifunctional, they may induce the clustering of their targets^14^, rendering the potential results somewhat questionable.

An alternative solution would be to investigate the behavior of tagged vesicle proteins. One widely used epitope tag is a pH-sensitive GFP variant, pHluorin^15^, which has been added to the luminal domains of multiple proteins of the synaptic vesicle (for example^5,9,16,17^) without modifying their functional properties in a fundamental fashion (for example^18^). The pHluorin molecules are quenched in the synaptic vesicle, due to the acidic environment found here, and become more strongly fluorescent upon exocytosis. In principle, this should enable their investigation with high precision, which should solve the issue of the vesicle protein behavior after exocytosis. A substantial problem, however, is that the pHluorin molecules have been analyzed in conventional, diffraction-limited microscopy in multiple studies, but their spectral characteristics preclude their use in techniques offering substantially higher resolution, such as stimulated emission depletion (STED) microscopy^19^ or photoactivated localization microscopy (PALM^20^).

Here we decided to tackle this problem by detecting the pHluorin moieties using affinity reagents that can be conjugated to fluorophores suitable for super-resolution microscopy. To avoid the potential clustering of molecules induced by antibodies, we relied on camelid-derived single-domain antibodies, which are typically termed “nanobodies” in the literature. These molecules are smaller than antibodies (molecular weights of ~12–15 KDa, as opposed to ~150 KDa for antibodies^21^), and are monovalent, which renders them preferable to antibodies. GFP nanobodies have been selected for several years (for example^22,23^) and pHluorin, as a GFP variant, should be an excellent target for all currently-available GFP nanobodies. We therefore employed GFP nanobodies to analyze two well-characterized pHluorin constructs, synaptobrevin-pHluorin (spH) and synaptophysin-pHluorin (sypHy; see for example^5,9,17^). We found that the nanobodies could be easily used for STED imaging of pHluorin molecules, under various stimulation conditions. Overall, the molecules were typically found in clusters larger and more intense than single pHluorin molecules.

## Results

### GFP nanobodies reveal pHluorin molecules in cultured neurons

**Figure 1:**
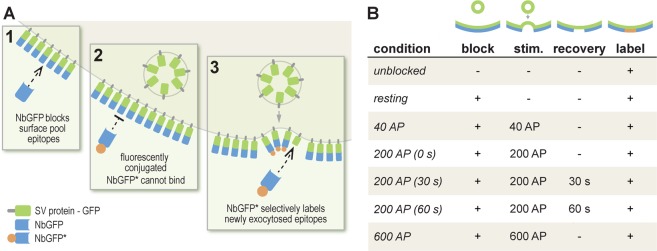
GFP nanobodies enable the selective labeling of newly-exocytosed vesicle proteins. (**A**) Neurons transfected with synaptic vesicle proteins coupled to pHluorin (a variant of GFP) on their luminal domains are incubated with non-fluorescently-conjugated single domain antibodies (typically referred to as nanobodies) directed against GFP (GFPNb; 1). This results in the surface epitopes of GFP becoming inaccessible to fluorescently-conjugated nanobodies, GFPNb* (2). Upon stimulating exocytosis, new vesicle proteins are exposed on the plasma membrane and are revealed by GFPNb* (3). (**B**) The table describes the conditions used here. The GFPNb* were applied for 10 seconds, either immediately following stimulation (0 s), or at the indicated time after stimulation (30 s, 60 s). The nanobody incubation was performed in divalent-free buffer, to reduce endocytosis.

Nanobodies have been used for several years to detect GFP-tagged proteins (for example^23^). Their main advantage over antibodies is the smaller size (~2 nm in diameter), which enables them to penetrate more easily into samples, and to detect more epitopes^24^. In addition, they cannot cross-link proteins, as they are monovalent, which should render them minimally disruptive to synaptic processes. Therefore a variety of experiments could be imagined, with various incubation and stimulation conditions. Here we decided to target the events taking place after synaptic vesicle exocytosis, through the following protocols (Fig. 1). First, to analyze the surface-resident pool of molecules we applied fluorescently-conjugated nanobodies to the living neurons for 10 seconds (in the absence of divalents, to inhibit endocytosis). To reveal selectively the newly exocytosed molecules, we adjusted this protocol by first incubating the neurons with saturating amounts of non-fluorescently-conjugated nanobodies, which blocked all of the surface-resident pool, before stimulating the cells and exposing them to fluorescently-conjugated nanoboodies (Fig. 1A). The stimulation paradigm ranged from 200 to 600 AP, at 20 Hz, in protocols that have been used in the past to release a significant proportion of the recycling pool (200 AP), or all recycling vesicles (600 AP), respectively^9,25^. We also used a 40 AP (20 Hz) protocol, which has been employed to stimulate the exocytosis of the readily releasable pool of vesicles^9,25^.

**Figure 2:**
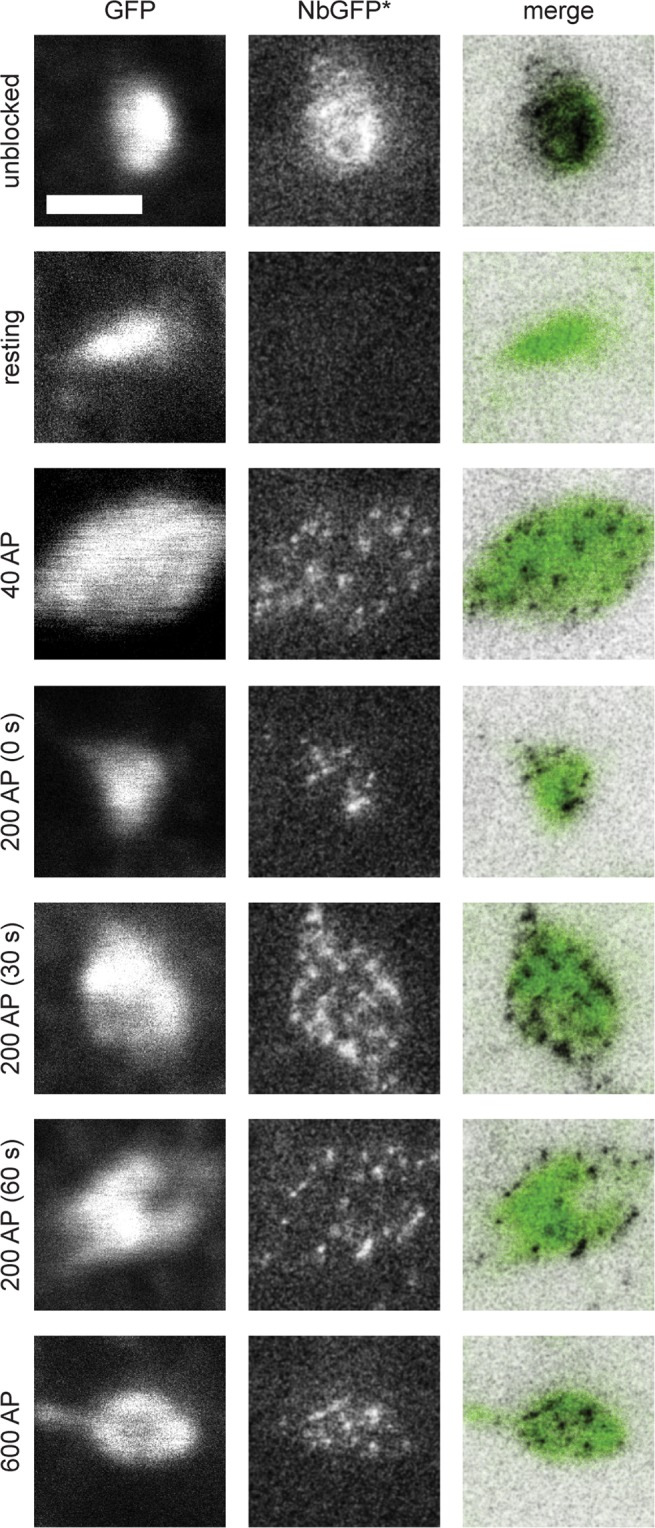
Labeling of newly-exocytosed sypHy molecules. We transfected neurons with sypHy and blocked their surface GFP epitopes by incubation with GFPNb for five minutes (37 °C) in Tyrode lacking Mg^2+^ or Ca^2+^. This was followed by stimulation in normal Tyrode (following the different protocols outlined in Fig. 1). The neurons were washed immediately after labeling with Tyrode lacking Mg^2+^ or Ca^2+^, and were fixed. The images show typical synaptic boutons. Left panels: confocal images of GFP. Middle panels: STED images of GFPNb*. Right panels: overlays. STED were processed by applying a 1-pixel Gaussian blur, to reduce the noise for optimal viewing, and they are inverted for the generation of the merged images. Scale bar 1 μm.

We first focused on mature hippocampal neuron cultures (see Methods), which were transfected with sypHy. As observed by many investigators in the past, this construct was expressed almost exclusively in synapses. The simple addition of nanobodies to the neurons resulted in ample synaptic staining (Fig. 2, top panels), which could be detected in both confocal and STED microscopy. We relied here on Atto647N, a red fluorophore that performs well in STED microscopy. The fluorescence intensity observed was substantially above background levels (Supplementary Fig. 1) and was reduced to undetectable levels by a preceding incubation with non-fluorescently-conjugated nanobodies (GFPNb; Supplementary Fig. 1). We then proceeded to stimulate the cultures, relying on trains of 200 AP. We first applied the fluorescently-conjugated nanobodies (GFPNb*) immediately after the stimulation train, for 10 seconds. The nanobody incubation was followed by fixation, and enabled the detection of multiple “spots” in STED microscopy, which were organized around the synaptic area, which was identified by monitoring the general pHluorin fluorescence. The nanobodies appear to need only a few seconds for accurate labeling, since increasing the incubation times from 10 to 120 seconds did not result in significant increases in nanobody fluorescence (Supplementary Fig. 2). Applying the nanobodies at different time points after stimulation, to detect the molecules that were still present on the plasma membrane, again revealed multiple “spots”, as did a longer stimulation train (600 AP; Fig. 2), or a 40 AP train.

To test whether the spots we observed were indeed representative of surface-stranded molecules, or whether they were already internalized, we washed the live neurons, after labeling, with a low pH solution, to strip the anti-GFP nanobodies from their epitopes. Nanobodies found on the surface would be removed, but nanobodies from endocytosed synaptic vesicles would not be affected. The experiments (shown in the Supplementary Fig. 3) showed that essentially all nanobodies were removed by this procedure, indicating that a negligible proportion had undergone endocytosis.

Overall, these results suggest that the GFP nanobodies readily detect pHluorin molecules on the plasma membrane.

### Synaptobrevin-pHluorin (spH) is detected both in synapses and in axonal areas

While sypHy was detected almost exclusively in areas resembling synaptic boutons, synaptobrevin-pHluorin (spH) is present both in synapses and on the plasma membrane of the axon^26,27^. The fraction of the molecules on the synaptic surface membrane is substantially lower than that present in synaptic vesicles (~20% in our hands^5^). The axonal population, outside of synapses, should be even lower than the synaptic surface fraction.

**Figure 3:**
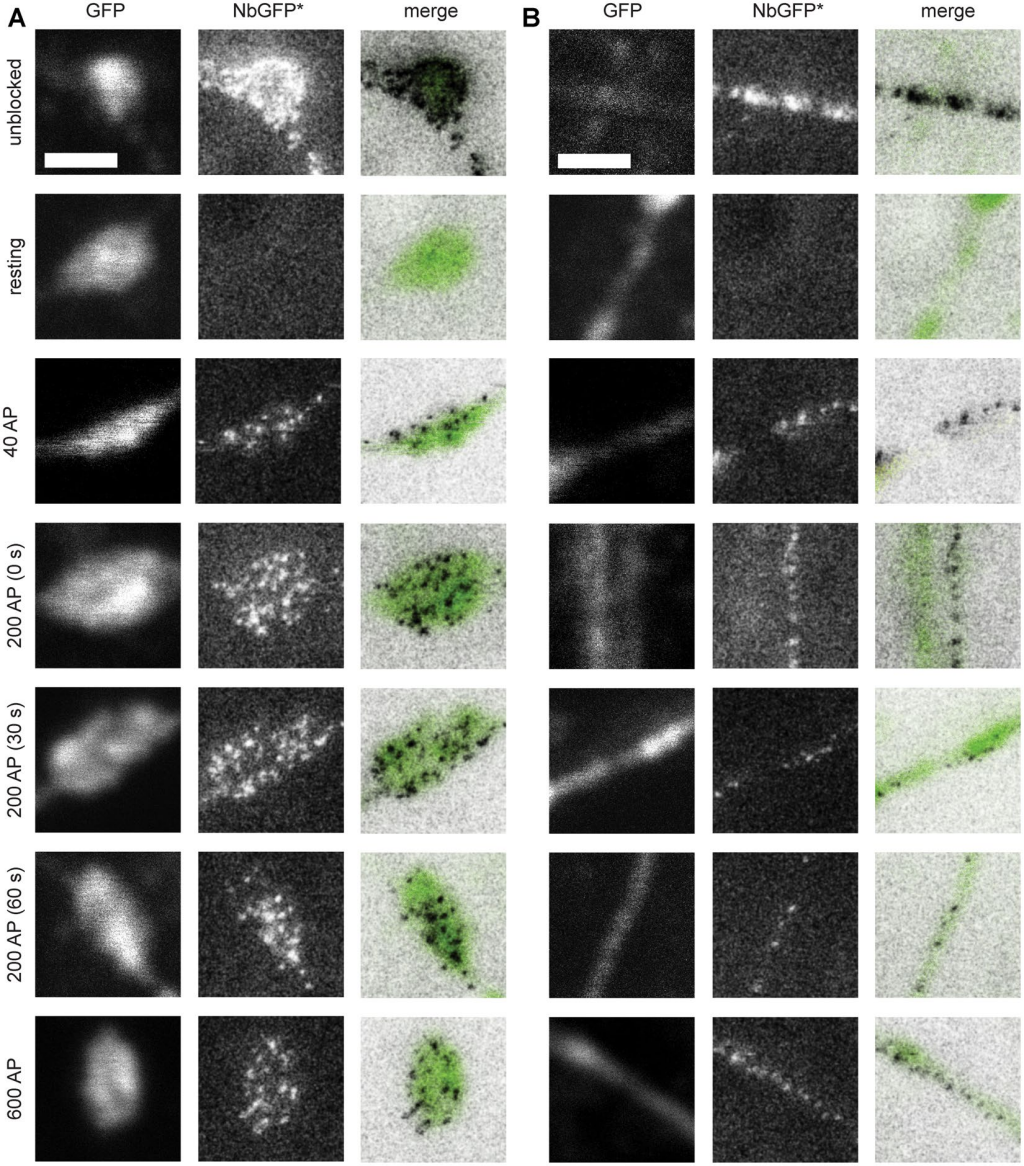
Visualization of newly-exocytosed vesicles in spH-expressing neurons. The same experiments as in Fig. 2 were performed in neurons transfected with spH. (**A**) Typical examples of stained synapses. (**B**) Typical examples of stained axonal areas. Left panels: GFP signal in confocal mode, middle panels: STED images of GFPNb*. Right panels: merge. As in Fig. 2, STED images are presented after application of a 1-pixel gaussian blur, and they are inverted for the generation of the merged images. Scale bar 1 μm.

To test whether the nanobody detection is sufficiently sensitive to reveal target molecules in axons, we expressed spH in hippocampal neurons, and we then stained the neurons using the same protocols as described above for sypHy. SpH molecules were revealed both in synapses (Fig. 3A) and in areas that, due to their morphology, were identified as axonal regions (Fig. 3B). Blocking the surface epitopes with non-fluorescently-conjugated nanobodies was as effective as for sypHy (Fig. 3). Stimulation resulted in the re-appearance of “spots” representing spH molecules, both in synapses and in axonal regions (Fig. 3).

### An analysis of pHluorin distribution

**Figure 4:**
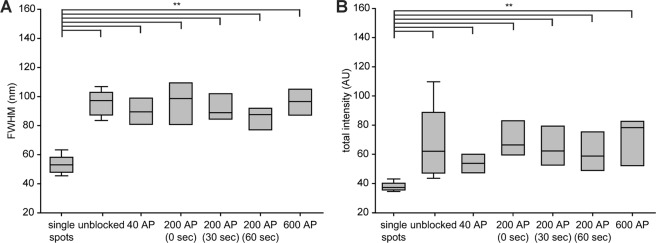
An analysis of sypHy spots. (**A**) The full width at half maximum, FWHM, of the sypHy spots was obtained from Lorentzian fits to the data obtained from sypHy-expressing neurons, and the mean values of individual experiments are shown here as box plots. We analyzed the differences between the different experimental values and the FWHM of single nanobodies, using ANOVA tests followed by Mann Whitney post-hoc tests, with a Bonferroni correction for multiple testing. All sypHy conditions are significantly different from the control, with p < 0.01. (**B**) A similar analysis for the spot intensity provided identical results. N = 5 to 9 independent experiments per condition.

The images presented so far indicate that pHluorin molecules form “spots” after exocytosis, of various sizes and intensities. To test whether these are indicative of pHluorin clusters, or are within the range of single pHluorin molecules, we first analyzed single nanobodies (Supplementary Fig. 4), which served as a standard for single pHluorins. We measured for all spots the full width at half maximum (FWHM) and the cumulative fluorescence intensity, provided by a Lorentzian fit to the spot profile. We then compared the FWHM and intensity of the single nanobodies to those of the spots observed in sypHy stainings (Fig. 4). The FWHM and the fluorescence intensity of the single nanobodies were significantly smaller than those of the sypHy spots (Fig. 4A,B).

**Figure 5:**
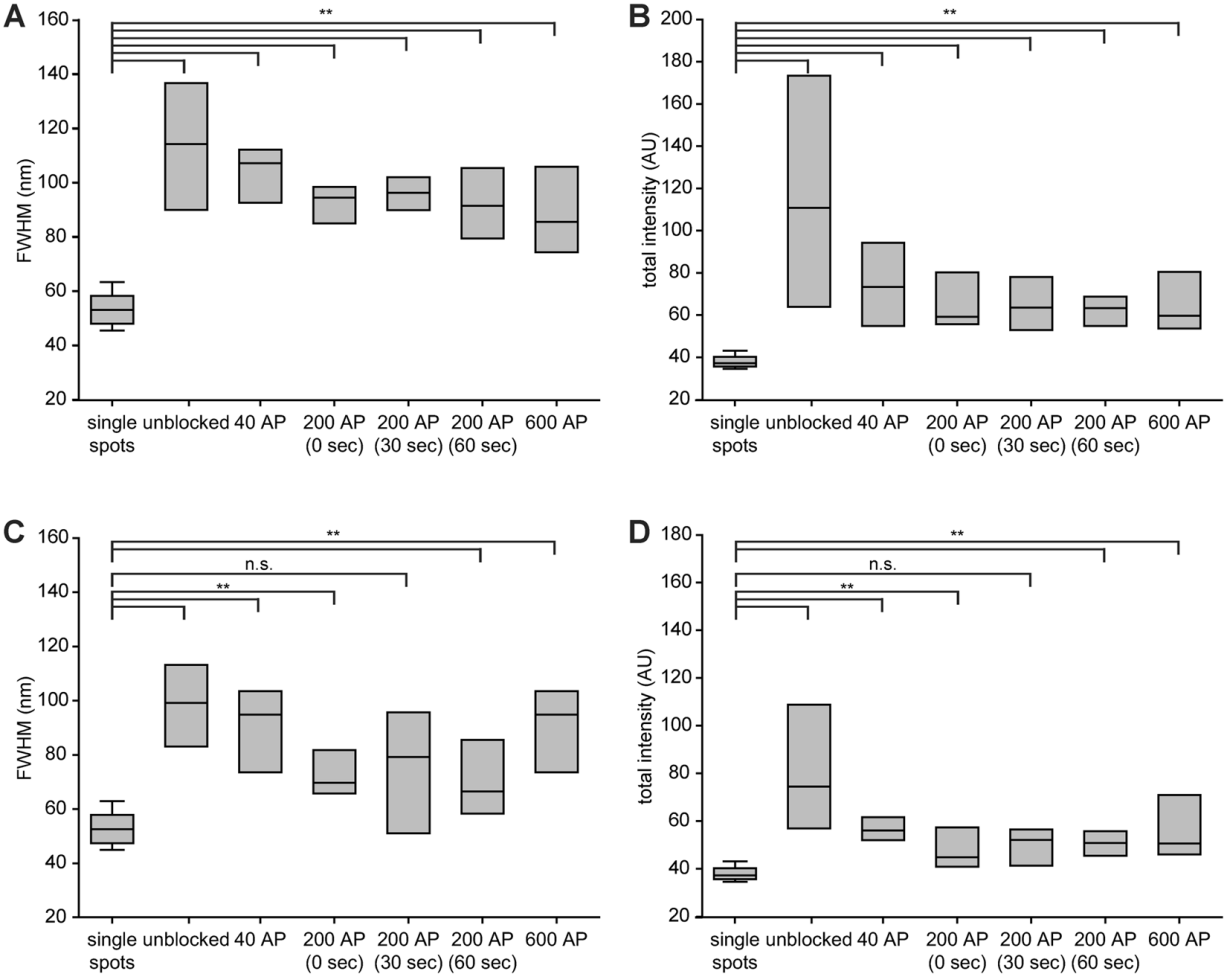
An analysis of spH spots. The FWHM of the spH spots was obtained from Lorentzian fits to the data in synapses (**A**) or in axons (**C**), as in Fig. 4. The same analysis as in Fig. 4 was performed. The conditions that are significantly different from the single nanobodies are indicated; **indicates p values smaller than 0.01. (**B**,**D**) show a similar analysis for the spot intensities. For synapses, N = 5 to 8 independent experiments per condition. For axons, N = 5 to 8 independent experiments per condition.

A similar result was obtained for spH in synapses (Fig. 5A,B), and also for spH in axons (Fig. 5C,D). Here also the spH population found at rest on the surface was organized in spots larger and brighter than single molecules, and this was the case for most conditions after stimulation as well.

### Co-expression with synaptophysin does not change substantially the spH pattern

One element that has been suggested in the past to control synaptobrevin and/or spH localization is the presence of synaptophysin, which interacts with synaptobrevin, and appears to target it to synaptic vesicles, while reducing its targeting to the axon^28^. We aimed to test the co-expression of the molecules here, to determine if the experimental procedure is successful under these conditions.

We co-expressed spH and synaptophysin coupled to mOrange (Supplementary Fig. 5), which is not detected by GFP nanobodies. Both proteins co-expressed well in neurons (Supplementary Fig. 5) and a reduction of the spH fluorescence in axons was apparent (Supplementary Fig. 6), with axons no longer as clearly lined by spH as in the case of single spH expression. We then stimulated the cultures, and analyzed them as in Figs 4–5. Overall, the results changed little in comparison to the spH expression (Supplementary Fig. 7), suggesting that nanobody-based pHluorin labeling is effective under these conditions as well.

### The nanobody detection of pHluorins can also be used in other neuronal preparations

To further test the applicability of the approach we introduced here, we tested it in a different preparation, the *Drosophila melanogaster* larval neuromuscular junction, relying on the well-studied synapses to the ventral 6 and 7 muscles^29^, of third instar larvae expressing spH^26^. The nanobodies revealed substantial signals in the synapses, which could be eliminated by blocking with non-fluorescently-conjugated nanobodies (Supplementary Fig. 8).

A STED analysis of the spH molecules found on the surface membrane indicated that at rest the molecules were organized in clusters, while after stimulation they tended to be found mostly as single molecules (Supplementary Fig. 9). This is not surprising, since this *Drosophila* line tends to shunt spH strongly to the plasma membrane^26^, possibly because this mammalian molecule is not well integrated within the *Drosophila* vesicles. Due to this issue, we did not pursue these experiments to the extent needed for biological claims on this preparation, as our purpose was only to test whether the GFPNb approach would be, in principle, applicable to this preparation.

## Discussion

Overall, our data suggest that one can circumvent the fact that the spectral characteristics of pHluorins are not optimally suitable for many super-resolution approaches, by staining them with nanobodies conjugated to flurophores designed for accurate performance in super-resolution. Using this approach we could analyze the organization of sypHy and spH molecules both at rest and after stimulation. SypHy molecules were always organized in spots larger and brighter than single molecules, which did not change substantially between stimulation conditions. The same was observed for spH molecules, both in synapses and in axons, in most of the conditions we tested.

These results are in line with a model in which there is a limited diffusion of molecules out of newly fused synaptic vesicles. This view is in line with multiple reports from the literature. For example, as indicated in the Introduction, immunostaining the luminal synaptotagmin 1 epitopes with antibodies has typically revealed spots that appeared larger and/or brighter than single antibodies or single synaptotagmin 1 molecules^5,8,9,30,31^. Since many synaptic vesicle proteins interact relatively strongly with each other^28,32–34^ and may even remain together upon detergent solubilization of the synapses^35^, it is perhaps not surprising that at least some are found to colocalize after exocytosis. Moreover, they may be stabilized by the endocytosis machinery immediately after exocytosis, which would only serve to strengthen this phenomenon^36^.

The opposite view, that synaptic vesicle proteins fully diffuse from each other upon exocytosis, has been initially been derived from pHluorin experiments in which the neuronal cultures were strongly stimulated. This resulted in the exocytosis of one pool of pHluorin molecules, followed by the endocytosis of a non-identical pool^25,37^ which has been interpreted at the time as evidence for an extensive intermixing of synaptic vesicle molecules on the plasma membrane, triggered by the separation of the molecules after exocytosis. An interesting experiment in this direction has been largely overlooked, however, from the work of Wienisch and Klingauf ^25^, who mildly stimulated pHluorin-expressing synapses, and observed that the pool endocytosed contained no previously exocytosed molecules. This implies that upon mild stimulation, within the physiological capacity of the cultured hippocampal neurons, newly exocytosed vesicles mix none of their molecules with those from the surface membrane. This model could not accommodate a complete separation of vesicle proteins in the plasma membrane upon exocytosis.

Our results, which analyze now this phenomenon in a more direct fashion, suggest that only a limited diffusion of pHluorin molecules needs to be taken into account. Some of the spH molecules were found in axons, in the form of clusters, but these formed only a minor fraction of all spots (~12%), which implies that the diffusion of spH to axons is quantitatively limited.

Among the caveats of our work, however, two need to be taken into account. First, ultrafast endocytosis^38^ may take place even before the nanobodies would be able to reach the synaptic cleft. These endocytosis events would be missed by the current applications of this technique. Other implementations, using more elaborate designs, including the pre-labeling of all pHluorin moieties, before stimulation, may ultimately also test this mechanism of endocytosis, but this is currently not tackled here.

Second, it is not clear how much the GFP moiety perturbs the vesicle molecules, and therefore how relevant the pHluorin behavior is for the native synaptic vesicle proteins. Substantial evidence points to the pHluorin effect being negligible. For example, a thorough test of the organization of GFP-tagged proteins versus their non-tagged variants revealed few differences^39^. Also, pHluorin-coupled synaptobrevin is known to function in exocytosis^18^. Nevertheless, testing the organization of native vesicle proteins with small monovalent probes will be necessary in the future. A number of proteins could serve as targets, albeit not synaptobrevin, since this molecule lacks an intraluminal domain. Proteins as synaptotagmin or the GABA transporter (VGAT), for which luminal domain antibodies have been established^10,40^, should form optimal targets, and may be investigated therefore in the near future.

## Methods

### Probes

#### Antibodies

For detection of bruchpilot, a mouse antibody (Catalogue number nc 82, *Drosophila* studies hybridoma database (DSHB), Iowa, US; deposited by E. Buchner) was used at a working dilution of 1:25, revealed by a Cy3-labelled goat Fab fragment against mouse (Catalogue number 115-167-003, Dianova, Terrebonne, QC, Canada), at a working dilution of 1:75.

#### Nanobodies

For the detection of pHluorins, we used a GFP nanobody coupled to Atto647N (FluoTag®-Q anti-GFP, from NanoTag Biotechnologies GmbH, Göttingen, Germany, Catalogue Number N0301-At647N-L) at a working dilution of 1:100. For blocking pHluorin surface epitopes, the same nanobody was used without the fluorophore, at a dilution of 1:20, unless otherwise indicated (FluoTag®-Q anti-GFP, NanoTag, N0300).

### Hippocampal cultured neurons

Cultured were generated from newborn rat pups (P1 or P2). The rat pups were decapitated, followed by dissection of the hippocampi, using conventional methods. The hippocampi were then placed in Hank’s balanced salt solution (HBSS) consisting of NaCl (140 mM), KCl (5 mM), glucose (6 mM), NaHCO_3_ (4 mM), Na_2_HPO_4_ (0.3 mM) and KH_2_PO_4_ (0.4 mM). The following enzyme mixture was added for one hour: 2.5 U/ml papain, in DMEM medium containing 0.5 mg/ml L-cysteine, 100 mM CaCl_2_, and 50 mM EDTA. Following this treatment, the enzymatic digestion was inactivated by adding albumin (0.2 mg/ml), trypsin inhibitor (0.2 mg/ml), and fetal calf serum (10%, volume/volume), for 15 minutes. The cells were separated by mechanical disruption, and were seeded at a concentration of 80,000/cm^2^, on circular coverslips (1.8 cm in diameter). The coverslips were coated with poly-L-lysine, after cleaning with nitric acid and sterilization. The neurons were then allowed to attach to the coverslips for 1–4 hours (at 37 °C) in DMEM containing 3.3 mM glucose, 2 mM glutamine and 10% horse serum. Afterwards they were switched to neuronal culture media, Neurobasal-A (with the additions of the B27 supplement at 2%, GlutaMax at 1%, and penicillin/streptomycin mixtures, at 0.2%). The cultures were maintained at 37 °C, under atmosphere with 5% CO_2_.

### Transfections

We transfected the cells as follows, relying on lipofectamine procedures. We mixed the plasmids with OptiMEM, using 1 μg of plasmid DNA for each coverslip to be transfected, in 25 µl OptiMEM. In parallel, we mixed 2 µl lipofectamine with an additional 23 µl OptiMEM. The mixtures were allowed to rest for 5 minutes, and were then combined, and were kept at room temperature for 20 minutes. The complete mixture was then applied to a coverslip, which was pre-incubated with DMEM containing 10 mM MgCl_2_ and 5 mM HEPES for 20–30 minutes. The coverslip was incubated with the transfection mixture for 20 minutes (37 °C), was washed twice with DMEM containing 10 mM MgCl_2_ and 5 mM HEPES, and was replaced in the original neuronal culture plate, in its original medium. Transfections were performed for 3–10 days before imaging.

### Fly husbandry and imaging techniques

We kept all flies (*Drosophila melanogaster*) at a constant temperature of 25 °C, and with a light/dark cycle of 12 hours. We used standard corn meal for all flies, replacing the food regularly at 14–21 days. We used the following lines: elavc155-gal4(x) (BDSC stock Nr. 458); uas-synaptopHlourin III^15^. For crossing flies, we placed virgin elavc155-gal4(x) females (five to ten per vial) together with uas-synaptopHlourin III males (also five to ten per vial). Dissections of fly larvae, to analyze the abdominal body wall muscles, were performed as previously described^29^, by pinning 3^rd^ instar larvae on Sylgard-coated dishes, cutting them along the midline (dorsally), and then eliminating all internal organs, including the basal ganglion. For imaging purposes the larvae cuticle was spread open as flat as possible.

### Electrical stimulation

We stimulated all preparations (hippocampal neurons and *Drosophila*) using an A310 AccupulserTM, which was driven through an A385 Stimulus Isolator (World Precision Instruments, Berlin, Germany). We used a platinum plate electrode, composed of two flat plates separated by 8 mm (produced in the machine shop of the Max Planck Institute for Biophysical Chemistry, Göttingen, Germany). The following buffers were used. For neuronal cultures, we relied on Tyrode, consisting of NaCl (124 mM), KCl (5 mM), MgCl_2_ (1 mM), CaCl_2_ (2 mM), HEPES (25 mM), glucose (30 mM), at pH 7.4. For *Drosophila*: NaCl (130 mM), KCl (5 mM), MgCl_2_ (2 mM), CaCl_2_ (2 mM), HEPES (5 mM), sucrose (36 mM), at pH 7.4. All stimulus trains are described in the main text.

### Blocking the surface epitopes, and labeling via GFP nanobodies

We blocked the surface epitopes in neurons by incubating the coverslips in 100 µl Tyrode containing GFPNb (but without MgCl_2_ and CaCl_2_). To minimize the amount of nanobodies used, the incubations were performed in a humidifying chamber, on Parafilm sheets, with the coverslip upside-down. Incubation times of 5 minutes were sufficient, at 37 °C. After a brief wash, the coverslips were subjected to stimulation, in normal Tyrode, and they were then immediately exposed to GFPNb* in 150 µl of Tyrode without MgCl_2_ and CaCl_2_. This was performed for 10 seconds. The coverslips were washed once with Tyrode (without MgCl_2_ and CaCl_2_) that had been pre-cooled on ice, and were fixed as explained in the Immunostaining section.

For *Drosophila* larvae, preparations were dissected as above, and were incubated with 100 µl GFPNb, for 10 minutes, in the buffer described under Electrical Stimulation. Electrical stimulation then followed after a brief wash, and the larvae were exposed immediately afterwards to GFPNb* (100 µl). This was incubated with the samples for two minutes. A brief wash was performed, before fixation and immunostaining.

### Immunostaining procedures

Fixation was performed using 4% PFA, with the addition of 0.2% glutaraldehyde. Fixation took place on ice (25 minutes for neurons, 30 minutes for *Drosophila*), and then at room temperature (20 minutes for neurons, 30 minutes for *Drosophila*). We then washed the samples 3x with PBS containing 100 mM NH_4_Cl and 100 mM glycine, to react with, and quench, all non-reacted fixative groups. After the last wash, this buffer was left on the samples for 20–30 minutes, for full quenching. Neurons were then blocked and permeabilized by incubation with PBS containing 1.5% BSA and 0.1% Triton X-100, for 15 minutes (with three solution changes). *Drosophila* preparations were blocked and permeabilized by a similar procedure, but with 0.5% Triton X-100, and for 30 minutes in total. We then applied the antibodies mentioned above. Every antibody incubation step was performed for one hour, in a humidifying chamber, at room temperature. Every antibody incubation step was followed by wash-off steps of 5–10 minutes, using the same buffers as for blocking. Three wash-off steps were always performed. After the last wash-off, the samples were exposed for 15–30 minutes (with three solution changes) to PBS containing 500 mM NaCl, and were finally washed two more times in PBS (10–20 minutes in total). Embedding or mounting then followed. In the case of *Drosophila*, large parts, such as the head and posterior end, were dissected away before embedding.

### Sample embedding or mounting

Neuronal cultures were embedded in Mowiol. This was prepared in-house by dissolving Mowiol (9.6%, weight per volume) in a buffer containing glycerol (24%, weight per volume) and Tris buffer (6 mM, pH 8.5). Embedding was performed in 8 µl Mowiol, on normal glass slides. *Drosophila* larvae were embedded in the same fashion, with the open (muscle-containing) side facing glass coverslips. We dried the samples at 37 °C for 40 minutes, before imaging, or by leaving them at room temperature overnight.

We also processed Drosophila samples by embedding in melamine, before cutting into 200 nm-thick sections (using an EM UC6 ultramicrotome; Leica, Wetzlar, Germany). Melamine solution was composed of 224 mg melamine (TCI, Chiyoda, Tokyo, Japan), with 8 mg p-Toluene, to which 96 µl water were added. Agitation on a rotating platform was applied until the solution dissolved completely (typically after approximately one hour). Larvae were covered with melamine solution droplets, on glass coverslips, with the open (muscle-containing) side facing the glass coverslips. We then placed an open (cut) BEEM capsule on the coverslips, and filled it with melamine solution to about 3 mm height. This was hardened at 40 °C for 24 hours, followed by filling the capsule completely with EpoFix resin, and an incubation of 72 hours at 60 °C. The coverslips were removed, and the melamin containing the larva was trimmed using a razor blade after the first 48 hours of incubation at 60 °C. Thin sections were then obtained using the ultramicrotome mentioned above, and they were mounted in Mowiol.

### Epifluorescence imaging

We imaged fixed samples using an Olympus IX71 microscope (Olympus, Hamburg, Germany), equipped with a TIRF objective (100x, 1.45 NA) from Olympus. We used a F-View II CCD camera (1376 × 1032 pixels; pixel width 6.45 μm). Alternatively, we used a normal 60x objective (1.35 NA, Olympus). Imaging filters were: for green fluorescence 480/40 HQ excitation, 527/30 HQ emission, 505 LP Q beam splitter (catalogue number F41-054; from AHF, Tübingen, Germany). For red fluorescence: 620/60 HQ excitation, 700/75 HQ emission, 660 LP Q beam splitter (catalogue number F41-054; from AHF, Tübingen, Germany).

For live imaging we used a Nikon Ti-E epifluorescence microscope (Nikon Corporation, Chiyoda, Tokyo, Japan), equipped with a Plan Apochromat objective (100x 1.4 NA oil-immersion, Nikon). Excitation was performed using a HBO-100W mercury lamp. We used a IXON X3897 camera (512 × 5 12 pixels; pixel width 16 μm; Andor, Belfast, Northern Ireland, UK). The microscope was climate-controlled using an OKOLab system (OKOLab, Ottaviano, Italy). Imaging filters were: for green fluorescence, excitation filter 470/40 nm, emission filter 525/50 nm and dichroic 495 nm. For red fluorescence: excitation filter 545/25 nm, emission filter 605/70 nm and dichroic 565 nm.

### STED and confocal imaging

We imaged all samples in STED and confocal mode using a TCS STED System (Leica Microsystems GmbH, Mannheim, Germany). This microscope was equipped with a HCX PL APO CS 100x objective (1.4 NA; oil immersion, Leica Microsystems). Excitation was performed using a 635 nm pulsed diode laser (PicoQuant, Berlin, Germany) for all STED imaging. STED depletion was performed using a Spectra-Physics MaiTai multiphoton laser at 750 nm (Newport Spectra-Physics, Santa Clara, CA, USA). Detection was performed using an Avalanche photodiode (Leica Microsystems). Confocal imaging was performed using the same microscope, relying on the 488 nm line of an Argon laser (for GFP), or a HeNe laser at 546 nm (for Cy3). Confocal detection was performed using photomultipliers.

### Image analysis

We analyzed all experiments using custom-written routines in Matlab (The Mathworks Inc., Natick, MA, USA). For epifluorescence quantification, we selected regions of interest (ROIs) containing synaptic boutons manually. We subtracted the average intensity of a manually-selected background region from the average intensities in the ROIs. This was performed for both the Atto647N and the GFP channels. The ratios of Atto647N fluorescence over GFP fluorescence were calculated independently for each bouton, and were reported in the respective figures. For STED imaging of GFPNb*, we first removed imaging noise by median filtering and average filtering. We then identified all spots above a threshold that was determined empirically. This provided the location of the spots, which enabled us to perform line scans on these locations (in the original raw data STED images), in the vertical and in the horizontal directions. The line scans were fit with Lorentzian functions, and the full width at half maximum (FWHM) and the fluorescence intensities (cumulative over the entire line scan) were quantified and were reported in the respective figures. The analysis methods reported here were largely derived from the PhD thesis of the first author, entitled “Quantitative Analysis of Synaptic Vesicle Membrane Trafficking”, and published in July 2018 at the University of Göttingen^41^.

## Supplementary information

Supplementary Figures

## Data Availability

The datasets generated during and/or analysed during the current study are available from the corresponding author on reasonable request.
