# Supplementary material for: GFP nanobodies reveal recently-exocytosed pHluorin molecules
Source: Sci Rep. 2019 May 23;9:7773. doi: 10.1038/s41598-019-44262-8 (PMC6533288; doi:10.1038/s41598-019-44262-8)
Supplement: Supplementary file 1 — Supplementary Figures [file 41598_2019_44262_MOESM1_ESM.pdf]

GFP nanobodies reveal recently-exocytosed pHluorin molecules

Katharina J. Seitz, Silvio O. Rizzoli

Supplementary Information

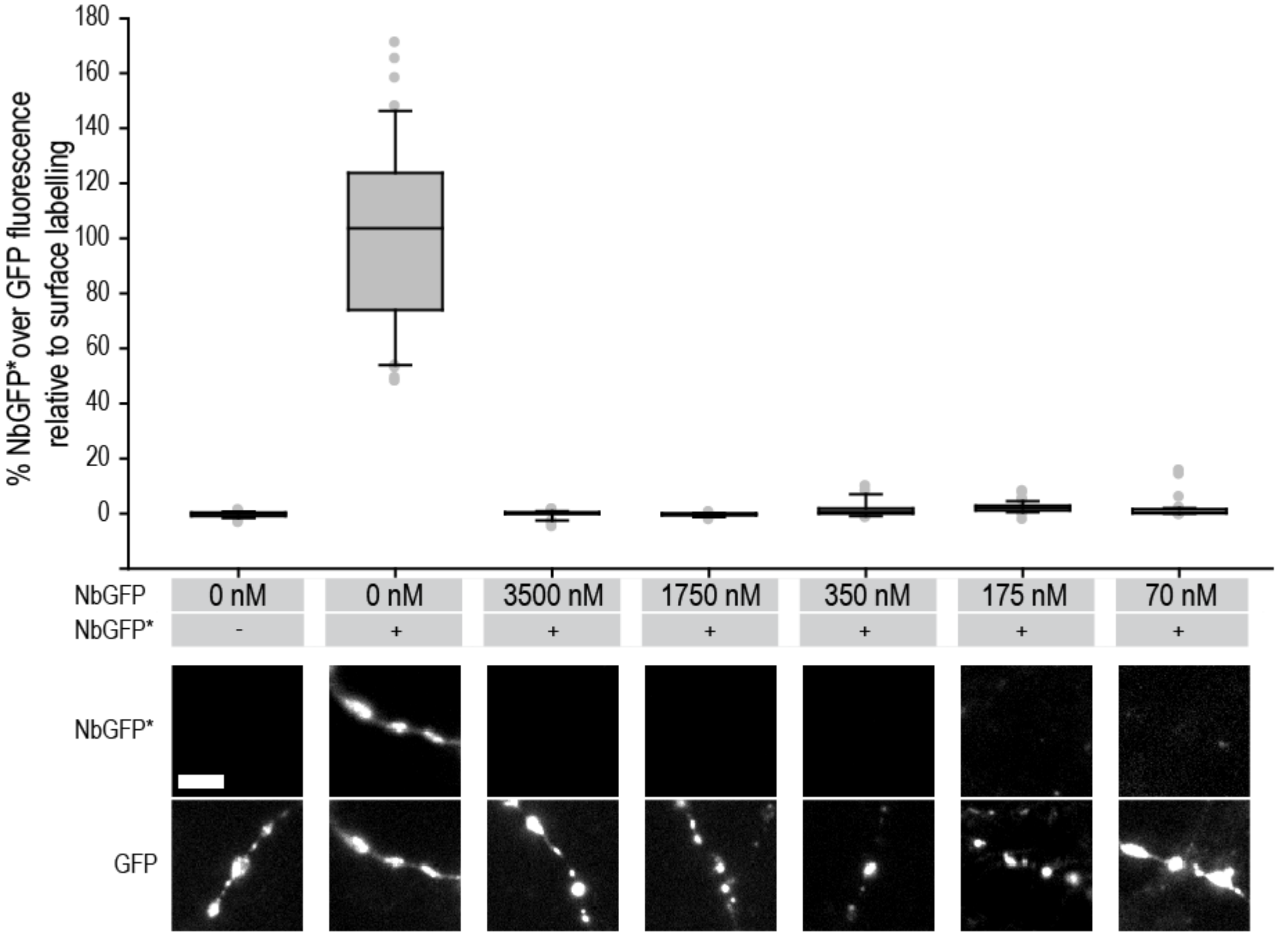

Supplementary Figure 1. Blocking pHluorin epitopes on the surface membrane. Neurons expressing sypHy were exposed to GFPNb for five minutes (as indicated in gray boxes), were then washed briefly, and were exposed to GFPNb*, for two minutes. The intensity of GFPNb* staining (Azzo647N), divided by the pHluorin intensity (GFP channel) was then measured, and was expressed in the graph, normalized to the control. N = 17 to 47 synapses, each from one experiment. Scale bar 5 µm. For clarity, this figure shows the results from one typical individual experiment. Multiple experiments were performed during the initial optimization stages, and the preferred blocking condition was then tested in every single experiment performed throughout this work (see for example the “resting” condition in Figures 2 and 3).

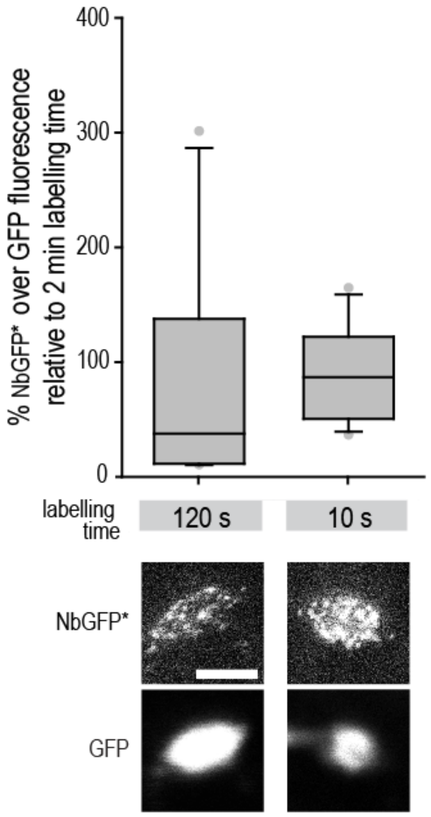

Supplementary Figure 2. Live staining of pHluorin molecules on the surface membrane. Neurons expressing sypHy were exposed to GFPNb*, for two minutes or for 10 seconds. The intensity of GFPNb* staining (Azzo647N), divided by the pHluorin intensity (GFP channel) was then measured, and was expressed in the graph, normalized to the control (the two-minute incubation). N = 10 to 12 synapses from one coverslip each. STED (GFPNb*) and confocal (GFP) images are shown as examples; scale bar 1 μm. For clarity, this figure shows the results from one typical individual experiment. Multiple experiments were performed during the initial optimization stages, and the preferred labeling condition, for 10 seconds, was then employed in every single experiment performed throughout this work (see for example the test conditions in Figures 2 and 3).

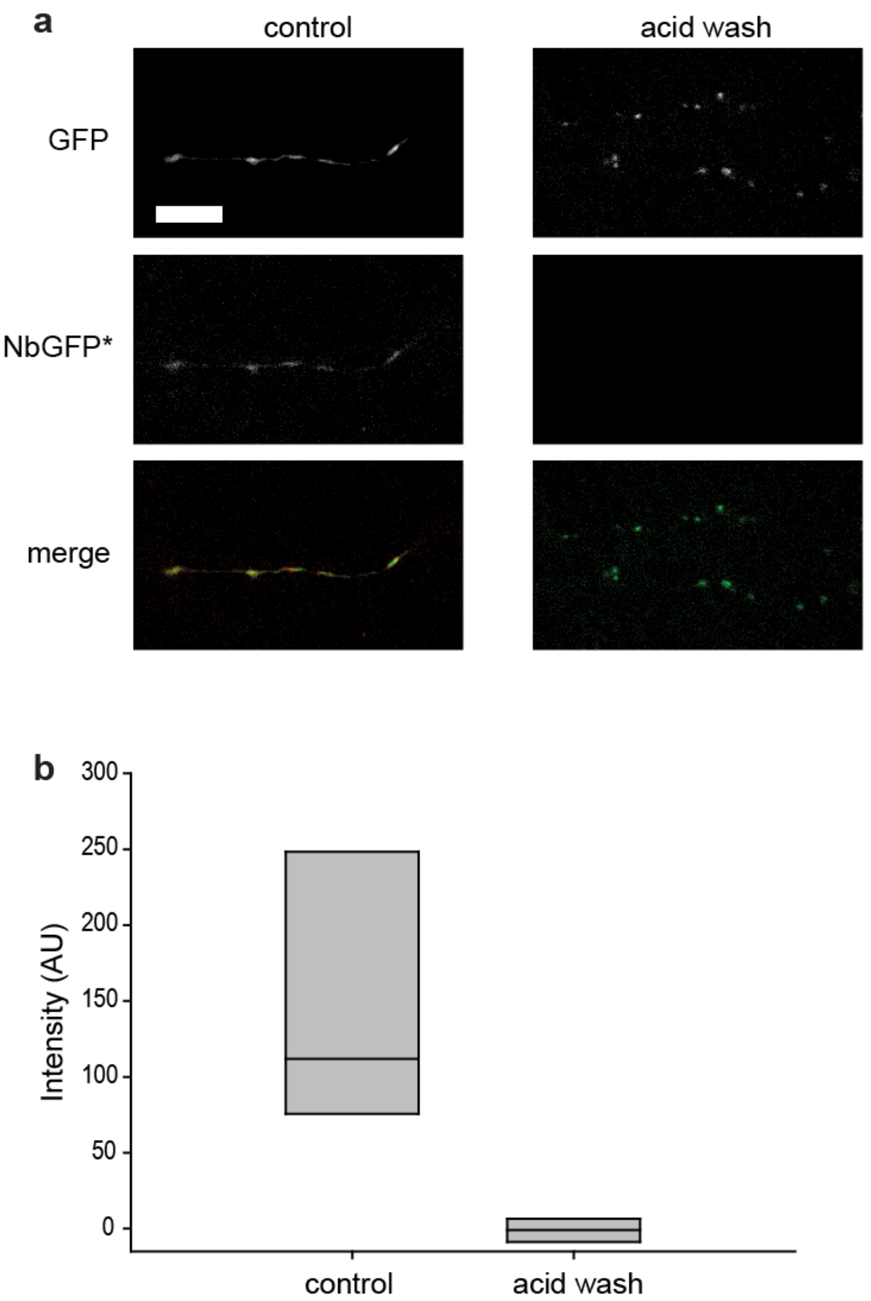

Supplementary Figure 3. Stripping surface-resident GFPNb*, to test for endocytosis effects. Neurons expressing sypHy were exposed to GFPNb for five minutes in divalent-free Tyrode, to block surface epitopes, followed by stimulation using 600 AP at 20 Hz. This was followed by labelling with GFPNb* for 10 seconds, immediately after stimulation, in divalent-free buffer. The cultures were kept for several minutes in the buffer, while imaging with an epifluorescence microscope, before washing three times with a freshly-prepared glycine buffer (150 mM glycine, pH 1.2, adjusted with HCl), followed by a 5-minute incubation period. The cultures were then returned to a neutral pH buffer (PBS), and were imaged again. (A) Control images, showing axonal processes from a live culture imaged before and after stripping. The pHluorin fluorescence is reduced in comparison to the control, since the low pH causes the unfolding of some of the GFP moieties, but is still visible. Scale bar, 10 µm. (C) Box plots analyzing the GFPNb* fluorescence intensity, before or after stripping. After stripping (acid wash) the GFPNb* fluorescence is indistinguishable from the background. N = 4 independent experiments, from two independent neuronal cultures.

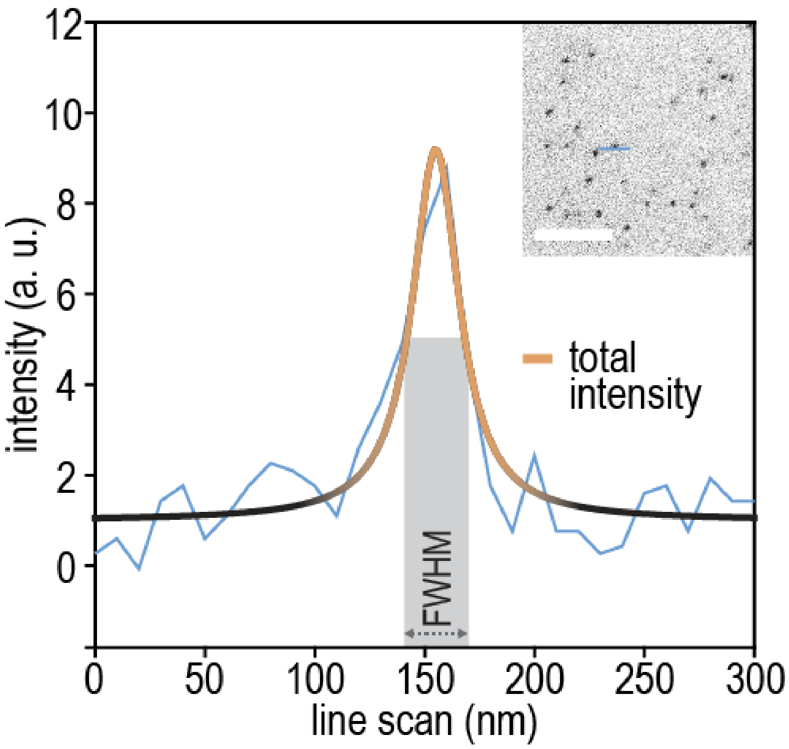

Supplementary Figure 4. Image analysis for single nanobodies. Line scans were drawn on STED spots (blue), and we then performed Lorentzian fits on individual STED spots. We determined from the fits the full width at half maximum (FWHM), which indicated the spot size, and also the total fluorescence intensity (cumulative, over the whole curve, subtracted for background). The inset shows GFPNb* diluted and placed on a coverslip, and then imaged in STED. Scale bar = 1 µm.

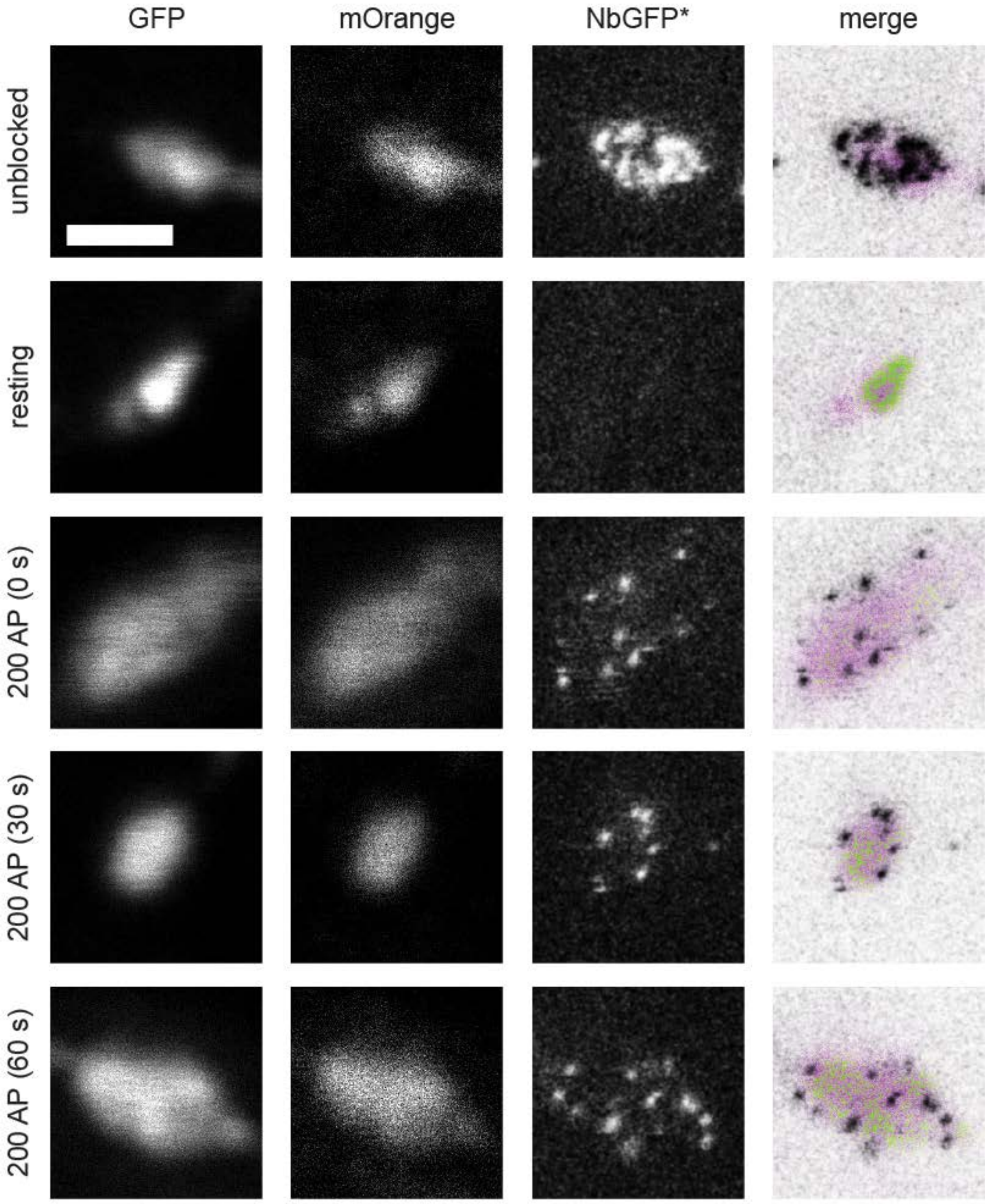

Supplementary Figure 5. Visualization of newly-exocytosed vesicles in spH- and synaptophysin-mOrange-expressing synapses. The same experiments as in Figure 2 were performed in neurons co-transfected with spH and synaptophysin-mOrange. Synapses are shown in this figure. Left panels: GFP signal in confocal mode, middle panels: mOrange signal in confocal mode, followed by STED images of GFPNb*. Right panels: merge. Images were treated as described in Figure 2. Scale bar = 1 µm.

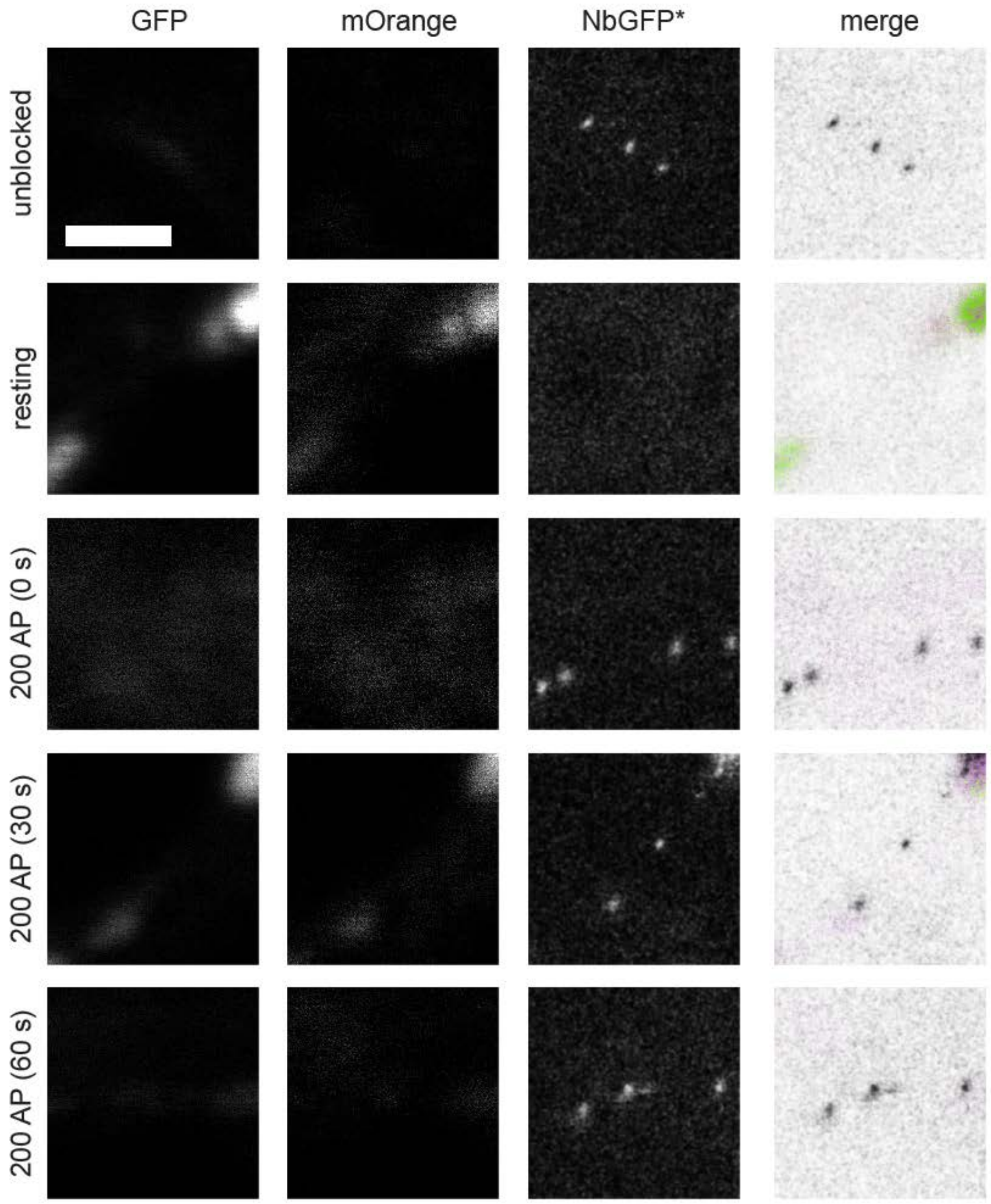

Supplementary Figure 6. Newly-exocytosed vesicle proteins in spH- and synaptophysin-mOrange-expressing axonal areas. The same experiments as in Supplementary Figure 4 were performed. Axonal areas are shown in this figure. Scale bar = 1 μm.

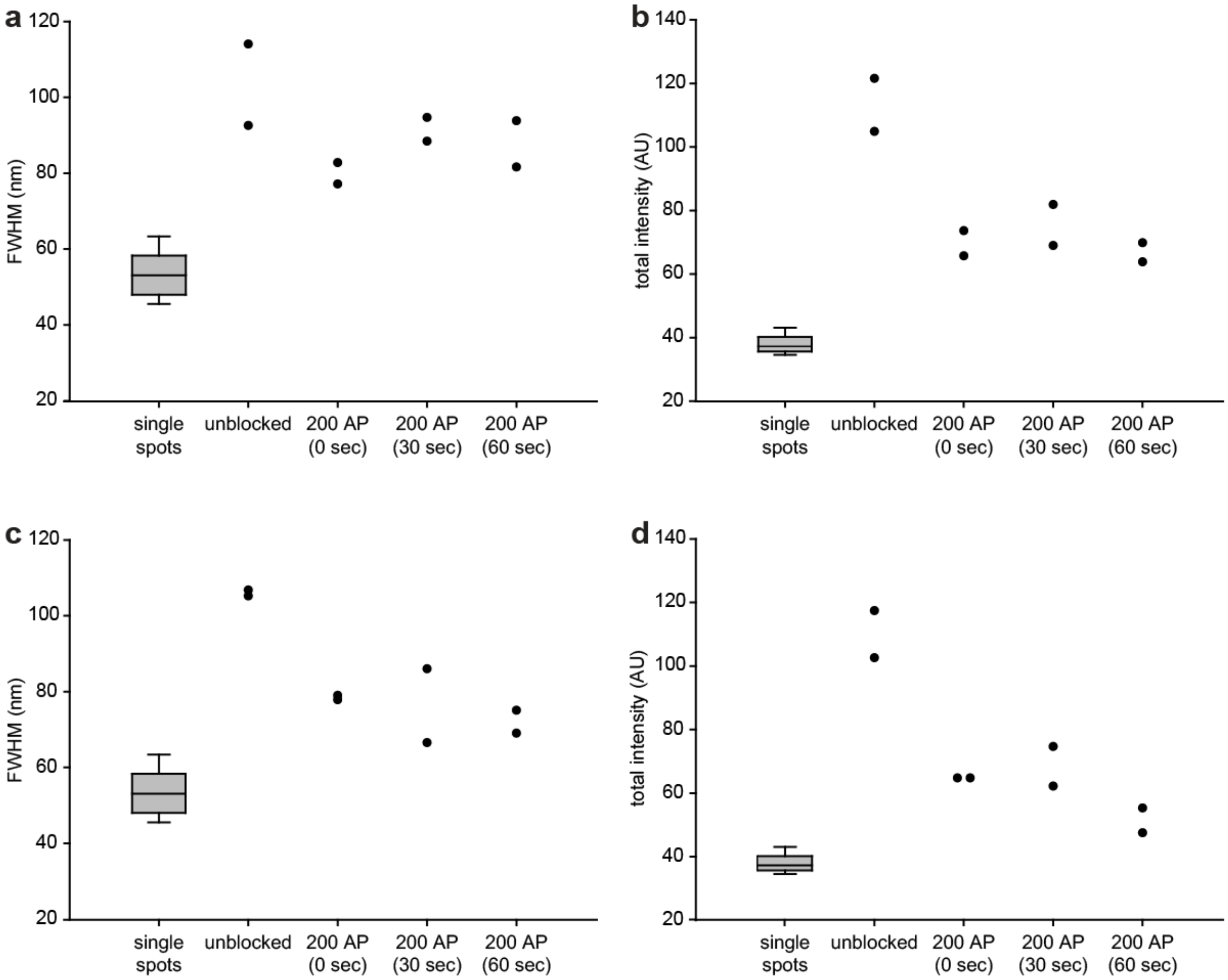

Supplementary Figure 7. An analysis of spH spots in neurons co-expressing spH and synaptophysin-mOrange. The FWHM of the spH spots was obtained from Lorentzian fits to the data in synapses (A) or in axons (C), as in Figure 4.; (B) and (D) show a similar analysis for the spot intensities. N = 2 independent experiments, with 45 to185 spots per condition for synapses, and 12 to 98 spots per condition for axons. The symbols show average values for the two independent experiments.

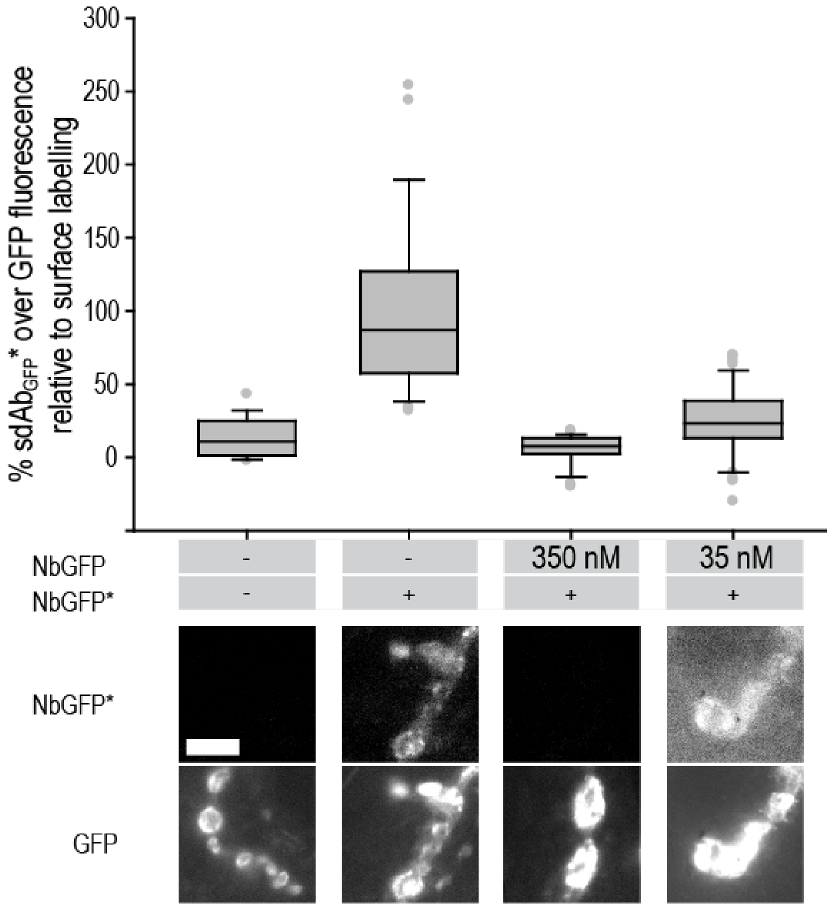

Supplementary Figure 8. Blocking of surface-resident GFP epitopes of *Drosophila* neuromuscular junctions expressing spH. Samples were exposed to GFPNb for 5, were washed briefly, and were stained with GFPNb* (two minutes). The intensity of GFPNb* staining (Azzo647N), divided by the pHluorin intensity (GFP channel) was then measured, and was expressed in the graph, normalized to the control. N = 17 to 45 synaptic boutons from 1 larva each. Epifluorescence images; scale bar 5 µm. For clarity, this figure shows the results from one typical individual experiment. Multiple experiments were performed during the initial optimization stages, and the preferred blocking condition was then tested in further experiments, such as those from Supplementary Figure 9. Nevertheless, these data remain qualitative in nature, and are only added here to indicate that this approach could be tried on *Drosophila* preparations.

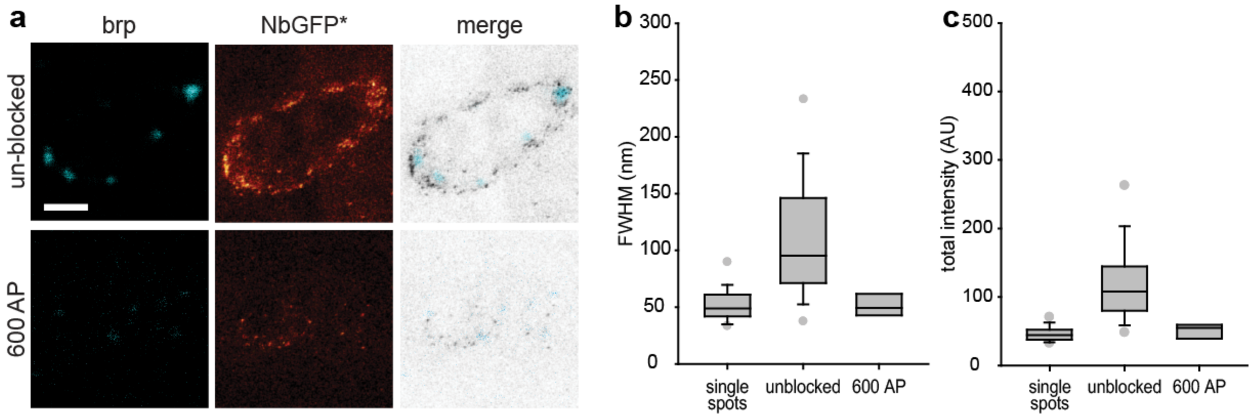

Supplementary Figure 9. Visualization of newly exocytosed vesicles in *Drosophila* neuro-muscular junctions. (A) STED images of spH epitopes labeled with GFPNb*, along with confocal images of bruchpilot (brp). Surface-labelling with GFPNb* was performed directly (un-blocked), or after blocking with GFPNb and after stimulation with 600 APs at 20 Hz. Fixation, immunostaining for brp, and embedding in melamine then followed. The samples were then cut in 200 nm sections, and were imaged. Scale bar = 3 µm. (B-C) The FWHM and the intensity of the STED spots were compared to those of single nanobodies. N = 18 (600 AP) to 119 spots (un-blocked) from one typical experiment.
